# Bacteremia due to *Acinetobacter ursingii* in infants: Reports of two cases

**DOI:** 10.11604/pamj.2016.23.193.8545

**Published:** 2016-04-15

**Authors:** Nurhayat Yakut, Eda Kadayifci Kepenekli, Ayse Karaaslan, Serkan Atici, Gulsen Akkoc, Sevliya Ocal Demir, Ahmet Soysal, Mustafa Bakir

**Affiliations:** 1 Division of Pediatric Infectious Diseases, Department of Pediatrics, Marmara University School of Medicine, Istanbul, Turkey

**Keywords:** Bacteremia, *acinetobacter ursingii*, child

## Abstract

*Acinetobacter ursingii* is an aerobic, gram-negative, opportunistic microorganism which is rarely isolated among *Acinetobacter* species. We present two immunocompetent infants who developed bacteremia due to *A. ursingii*. The first patient is a two -month- old boy who had been hospitalized in pediatric surgery unit for suspected tracheo-esophageal fistula because of recurrent aspiration pneumonia unresponsive to antibiotic therapy. The second patient is a fourteen -month- old boy with prolonged vomiting and diarrhea. *A. ursingii* was isolated from their blood cultures. They were successfully treated with ampicillin-sulbactam. Although *A. ursingii* has recently been isolated from a clinical specimen; reports of infection with *A. ursingii* in children are rare. *A. ursingii* should be kept in mind as an opportunistic microorganism in children.

## Introduction

*Acinetobacter* species are the most common causative agents of health care associated infections such as meningitis, ventilator-associated pneumonia, endocarditis and catheter-related bacteremia [1, 2]. Despite their low virulence, they have increasingly been recognized as opportunistic microorganisms particularly in hospitalized and immunocompromised patients. *Acinetobacter* species can be found widely in nature. The organism can be a part of the flora of the skin, the oral cavity and the upper respiratory, genital and gastrointestinal tracts. The skin may become a reservoir for *Acinetobacter* in hospitalized patients and health care provider [3]. Patients who have undergone a tracheostomy are at particularly high risk for colonization by *Acinetobacter*. Because they survive on inanimate surfaces for a long-term, these microorganisms can be commonly found in the hospital environment, particularly in moist areas such as in humidifiers and ventilators. Colonized medical equipment such as respiratory equipment and intravenous catheters may be responsible from nosocomial outbreaks [4]. Although *A. ursingii*, has recently been isolated from a clinical specimen, reports of infection with *A. ursingii* in children are rare [5]. Herein we report two cases with bacteremia due to *A. ursingii* in immunocompetent infants who were successfully treated.

## Patient and observation

### Patient 1

A two-month-old boy had been hospitalized in pediatric surgery unit for suspected tracheo-osefageal fistula. He was referred from another hospital with the diagnosis of recurrent aspiration pneumonia unresponsive to antibiotic therapy. On the admission, he had tachypnea and subfebrile fever on physical examination. Laboratory tests showed a white blood cell (WBC) of 10900/µl, C-reactive protein (CRP) of 11.8 mg/L and normal biochemistry values. Peripheral blood culture was obtained. Twelve hours later, the BacT/Alert device (bioMerièux, Marseille, France) gave a signal indicating a growth of microorganism. Gram staining was positive for gram-negative coccobacilli. The microorganism was nonmotile, strictly aerobic, catalase-positive and oxidase-negative. Empirical antibiotic treatment with meropenem and amikacin was started. The next day, Vitek MS (bioMerièux, Marseille, France) identified the microorganism as *Acinetobacter ursingii* and reliability was 99.9%. The E-test used to describe antimicrobiological susceptibility showed that the microorganism was susceptible to ampicillin-sulbactam, gentamicin, ciprofloxacin and imipenem. The antibiotic therapy was de-escalated to ampicillin-sulbactam. On the third day of the treatment, control peripheral blood culture was taken and remained sterile. He was successfully treated with ampicillin-sulbactam.

### Patient 2

A fourteen-month-old boy with the diagnosis of gastroesophageal reflux disease was admitted to our hospital with complaints of vomiting and diarrhea for a month. On admission, his physical examination revealed fever (38.4^°^C). Because of the prolonged diarrhea and vomiting, patient was hospitalized and further investigated. Laboratory tests showed a WBC of 5800/µl, CRP of 3.2 mg/L and normal biochemistry values. Peripheral blood culture was obtained and signaled growth for gram-negative bacilli. Empirical antibiotic treatment with meropenem and amikacin was started. Microorganism was identified as *Acinetobacter ursingii* by Vitek MS (bioMerièux) with a reliability of 99.9%. The e-test showed that *A. ursingii* was susceptible to ampicilin-sulbactam, gentamicin, ciprofloxacin and imipenem. The antibiotic therapy was deescalated to ampicin-sulbactam. On the third day of the treatment, control peripheral blood culture remained sterile. He was successfully treated with ampicillin-sulbactam.

## Discussion

*Acinetobacter ursingii* can cause life-threatening bloodstream infection especially in immunocompromised patients, but their correct identification is problematic. Currently, more than 32 genomic species have been identified by molecular methods [6, 7]. Virulence, epidemiology, antimicrobial susceptibility, clinical significance of *Acinetobacter* species may vary. Therefore, determining the prevalence of the *Acinetobacter* species causing invasive infection may be helpful in the management of infection [8, 9]. Risk factors for infection with *A. ursingii* emerged as noso-comial pathogens during the last 3 decades including underlying serious disease such as cancer, intravascular catheterization, treatment with broad spectrum antibiotics, prolonged hospitalization. Due to their ability for long-term survival in the environment, *A. ursingii* may spread among patients and cause nosocomial infections and outbreaks. Although *A. ursingii* have low pathogenic potential, it has increasingly been described as opportunistic pathogen particularly in immunosupressed and patients in intensive care units [10]. *Acinetobacter* may cause suppurative infection such as peritonitis, endocarditis, osteomyelitis, arthritis, pancreatic and liver abcesses. Clinical manifestations are similar to other bacterial infections and may vary depending on the serious underlying disease and immune status. *A. ursingii* related bloodstream infection may occur as primary or secondary to respiratory or urinary tract and wound infection. Immunosupressed neonates are at particularly high risk for primary bacteremia, and clinical signs may vary ranging from an asymptomatic to septic shock and disseminated intravascular coagulation. Because of increasing antibiotic resistance, treatment of infections due to *Acinetobacter spp*. has become difficult. Antibiotic treatment should be determined by in vitro susceptibility testing. *A. ursingii* seems to be more susceptible to antimicrobial agents compared to *A. baumannii*. Infections due to *A. ursingii* are rare in healthy children. There are a few reports of infection with *A. ursingii* in the literature; most of the cases are immunocompromised with underlying serious disease. Report of a bacteremia caused by *A. ursingii* in an adult patient with oro-pharyngeal cancer was reported [11].

## Conclusion

Although *A. ursingii*, has recently been isolated from a clinical specimen, reports of infection with *A. ursingii* in children are rare. Our patients had neither of central venous catheter, immuno-compromised status, severe underlying co-morbidities and prolonged hospitalization. Source of *A. ursingii* may either be the hospital or colonization prior to admission. As a conclusion, although rare, *A. ursingii* should be kept in mind as an opportunistic microorganism in children.

## References

[1] Chuang YC, Sheng WH, Li SY (2011). Influence of genospecies of Acineto-bacterbaumannii complex on clinical outcomes of patients with Acinetobacter bacteremia. Clin Infect Dis.

[2] Turton JF, Shah J, Ozongwu C (2010). Incidence of Acinetobacter species other than A baumannii among clinical isolates of Acinetobacter: evidence for emerging species. J ClinMicrobiol.

[3] Seifert H, Dijkshoorn LP, Gerner-Smidt P (1997). Distribution of Acinetobacter species on human skin: comparison of phenotypic and genotypic identification methods. J Clin Microbiol.

[4] Kilic A, Li H, Mellmann A (2008). Acineto-bactersepticus sp nov association with a noso-comial outbreak of bacteremia in a neonatal intensive care unit. J Clin Microbiol.

[5] Nemec A, De Baere T, Tjernberg I (2001). Acineto-bacterursingii sp nov and Acineto-bacterschindleri sp nov, isolated from human clinical specimens. Int J Syst Evol Microbiol.

[6] Karah N, Haldorsen B, Hegstad K (2011). Norwegian Study Group of Acinetobacter: species identification and molecular characterization of Acinetobacter spp blood culture isolates from Norway. J Antimicrob Chemother.

[7] Nemec A, Musílek M, Maixnerová M (2009). Acineto-bacterbeijerinckii sp nov and Acineto- bactergyllenbergii sp nov, haemolytic organisms isolated from humans. Int J Syst Evol Microbiol.

[8] Wisplinghoff H, Paulus T, Lugenheim M (2012). Nosocomial bloodstream infections due to Acineto-bacterbaumannii, Acinetobacterpittii and Acinetobacternosocomialis in the United States. J Infect.

[9] Lee SY, Shin JH, Park KH (2014). Identification, genotypic relation, and clinical features of colistin-resistant isolates of Acinetobacter genomic species 13BJ/14TU from bloodstreams of patients in a university hospital. J Clin Microbiol.

[10] Van den Broek PJ, Van der Reijden TJ, Van Strijen E (2009). Endemic and epidemic acinetobacter species in a university hospital: an 8-year survey. J Clin Microbiol.

[11] Endo S, Sasano M, Yano H (2012). IMP-1-producing carbapenem-resistant Acineto-bacterursingiifrom Japan. J Antimicrob Chemother.

